# Gallic acid as a biointeractive dentin conditioning agent for universal adhesives: bond strength, iron retention, and surface characterization following ferric sulfate hemostatic contamination

**DOI:** 10.1007/s00784-026-06987-5

**Published:** 2026-06-26

**Authors:** Nasibe Aycan Yılmaz

**Affiliations:** Department of Restorative Dentistry, Faculty of Dentistry, Dokuz Eylül University, İzmir, Turkey

**Keywords:** Gallic acid, Universal adhesives, Dentin contamination, Dentin adhesion, Restorative dentistry, Ferric sulfate

## Abstract

**Objectives:**

This study evaluated the effects of two universal adhesives, applied under three protocols, self-etch (SE), conventional total-etch (CTE), and modified total-etch (MTE) with 1% (w/v) gallic acid, on shear bond strength (SBS), iron retention, and surface characteristics of noncontaminated and ferric sulfate-contaminated dentin.

**Materials and methods:**

One hundred eighty dentin specimens prepared from ninety extracted human molars were allocated to 12 groups (*n* = 15) by adhesive type (All Bond Universal [ABU] or Tokuyama Universal Bond [TUB]), application protocol (SE, CTE, or MTE), and contamination condition (noncontaminated or 20% ferric sulfate-contaminated [ViscoStat]. After composite build-up and 5,000x thermocycling, SBS was measured. Surface morphology (*n* = 3, 6 groups) and elemental composition (*n* = 5, 5 groups) were characterized by scanning electron microscopy (SEM) and energy-dispersive X-ray spectroscopy (EDS). SBS data were analyzed by three-way ANOVA (α = 0.05).

**Results:**

All three variables significantly affected SBS (*p* < 0.001). SE yielded the lowest SBS values (17.46 ± 3.19 MPa) among all protocols (*p* < 0.001). CTE and MTE protocols showed comparable SBS values (20.30 ± 3.20 vs. 20.80 ± 3.05 MPa; *p* = 0.591). After ferric sulfate contamination, CTE produced no detectable iron residue, whereas MTE retained iron-associated surface deposits. EDS revealed descriptively higher mineral content in gallic acid-conditioned dentin, and SEM showed protocol-dependent surface patterns.

**Conclusions:**

Gallic acid yielded short-term bond strength comparable to phosphoric acid, while inducing a more conservative surface modification. However, gallic acid conditioning following ferric sulfate contamination should be regarded cautiously, as iron-associated deposits are preserved.

**Clinical relevance:**

Gallic acid may be considered a conservative preconditioning option for noncontaminated dentin. After ferric sulfate contamination, phosphoric acid remains the more predictable choice given its iron-removal capacity.

**Supplementary Information:**

The online version contains supplementary material available at 10.1007/s00784-026-06987-5.

## Introduction

The long-term clinical performance of resin-based restorations depends critically on the quality of adhesive bonding to dentin, a substrate inherently susceptible to contamination, enzymatic degradation, and interfacial failure over time [1, 2]. Although rubber dam isolation remains the standard of care, certain clinical scenarios, including subgingival caries, malpositioned teeth, or limited patient cooperation, render adequate isolation challenging, making the development of conditioning agents that ensure reliable bonding while preserving dentin mineral content a recognized research priority [1].

Conventional total-etch (CTE) protocols employing 35–40% phosphoric acid effectively remove the smear layer but cause aggressive demineralization extending up to 10 μm into dentin [2–4], increasing substrate permeability and activating endogenous matrix metalloproteinases (MMPs), potentially compromising long-term bond durability [5–8]. Consequently, using conventional mild self-etch (SE) adhesives or universal adhesives applied with SE approaches [2, 9, 10] is often advocated to minimize these adverse effects. In contamination-prone scenarios, phosphoric acid preconditioning is recommended due to its decontaminating capacity [11–16]. Nevertheless, alternative conditioning agents capable of effective decontamination while minimizing excessive demineralization remain limited [8, 17].

Contemporary universal adhesives offer clinical versatility through their multi-mode application capability; however, their performance is influenced by both the selected etching protocol and substrate condition [2, 18, 19]. Evidence regarding the performance of universal adhesives under contaminated conditions remains incomplete. Existing studies have predominantly focused on single adhesive systems or limited contamination scenarios [1, 14, 16, 20, 21]. leaving the combined influence of conditioning protocol selection and adhesive composition on bonding outcomes following hemostatic agent exposure insufficiently characterized [17].

As restorative dentistry advances toward minimally invasive, biologically driven approaches, the development of conditioning agents that preserve dentin mineral content while ensuring reliable adhesive bonding has emerged as a research priority. Among candidate biointeractive conditioning agents, gallic acid (3,4,5-trihydroxybenzoic acid), a plant-derived polyphenol with a well-characterized capacity to interact with both organic and inorganic dentin components, merits systematic investigation. Its multimodal activity profile encompasses collagen-stabilizing and cross-linking capacity through hydrogen bonding with polypeptide chains [22–25], antioxidant [26] and antibacterial properties [27–29], inhibition of enamel demineralization [30] and remineralization of enamel [31], Ca²⁺ chelation activity [32, 33], and metal-chelating properties, including strong complexation with Fe³⁺ [34–37]; furthermore, gallic acid has demonstrated MMP-inhibitory activity [23, 25, 38]. This constellation of biological interactions aligns with the emerging paradigm of dentin-interactive agents that seek to complement mechanical adhesion with biological substrate protection. A modified total-etch (MTE) protocol employing 1% (w/v) gallic acid as a discrete preconditioning step prior to universal adhesive application has been previously described in the literature [17]. In this MTE protocol, the 1% (w/v) gallic acid solution is applied to the dentin surface with a microbrush in a scrubbing motion, rinsed thoroughly, and dried prior to adhesive application, functioning as a polyphenol-mediated conditioning agent distinct from conventional phosphoric acid etching in both chemical mechanism and demineralization depth. To the best of the present author’s knowledge, this specific application strategy was first evaluated by Goncu and Yilmaz (2022), who employed Single Bond Universal adhesive under both noncontaminated and hemostatic agent (Ankaferd Blood Stopper)-contaminated dentin conditions. The authors reported significantly higher shear bond strengths following gallic acid preconditioning relative to both self-etch and conventional total-etch protocols, with the performance advantage most pronounced under contamination [17]. A subsequent investigation by Ghanbarian et al. (2025), conducted on noncontaminated dentin without artificial aging, demonstrated that 1% and 2% (w/v) gallic acid preconditioning prior to Gluma Bond Universal significantly increased shear bond strength relative to unmodified self-etch and total-etch controls, with the 1% concentration outperforming the 2% formulation in both adhesive strategies [39]. Notably, neither prior study evaluated ferric sulfate-based hemostatic contamination, nor did either incorporate elemental surface characterization of gallic acid-conditioned dentin.

The present study was designed as a direct extension of that prior investigation [17], with three substantive advances: (1) substitution of a ferric sulfate-based hemostatic agent for the Ankaferd Blood Stopper-based contamination model used previously, enabling iron-specific elemental characterization; (2) the addition of EDS elemental analysis and SEM morphological assessment, analytical dimensions absent from [17], to characterize gallic acid–iron surface interactions directly; and (3) a dual-adhesive comparison of two mechanistically distinct universal adhesive systems to determine whether adhesive composition modulates bonding performance under the tested conditions. The confirmatory dimension of the present study concerns the replication of adequate short-term bond strength following gallic acid preconditioning; its novel contributions include elemental characterization of iron retention, a ferric sulfate-specific contamination model, and a dual-adhesive design, two methodological dimensions directly addressed in the present investigation.

Gallic acid’s capacity to form stable complexes with Fe³⁺ has been exploited in tubule-occluding formulations for the management of dentin hypersensitivity [34–36]. Given that ferric sulfate-based hemostatic agents deposit iron residues on dentin surfaces, gallic acid’s strong affinity for Fe³⁺ suggests potential interaction with these contaminants. However, whether gallic acid removes residual iron from contaminated dentin or forms surface-bound gallic acid-iron complexes remains unclear, as does the impact of such interactions on adhesive performance. Two universal adhesives with distinct functional monomers and activation mechanisms were selected: All Bond Universal (ABU; Bisco Inc., Schaumburg, IL, USA), a light-cured, single-component system containing 10-methacryloyloxydecyl dihydrogen phosphate (10-MDP) as its primary functional monomer (pH = 3.2); and Tokuyama Universal Bond (TUB; Tokuyama Dental Corp., Tokyo, Japan), a self-cured, two-component system containing a three-dimensional self-reinforcing phosphoric acid monomer (3D-SR) and MTU-6 without 10-MDP (pH = 2.2). The differing functional monomers, polymerization mechanisms, and pH values of these systems may influence their interaction with dentin substrates and their susceptibility to contamination-related performance changes [18, 19]. The lower pH of TUB (2.2) relative to ABU (3.2) may furthermore influence calcium salt formation and monomer infiltration efficiency, particularly on mineral-preserved substrates. Therefore, this study primarily aimed to compare the shear bond strength of two universal adhesives (All Bond Universal [ABU] and Tokuyama Universal Bond [TUB]) on noncontaminated versus ferric sulfate-contaminated dentin using three application protocols: self-etch (SE), conventional total-etch (CTE), and modified total-etch (MTE) with gallic acid preconditioning. A secondary aim was to evaluate whether 1% (w/v) gallic acid modifies residual iron content on ferric sulfate-contaminated dentin surfaces, as characterized by EDS elemental analysis and SEM morphological assessment. Shear bond strength (SBS) testing, scanning electron microscopy (SEM), and energy-dispersive X-ray spectroscopy (EDS) were employed to evaluate mechanical performance, surface morphology, and residual elemental composition, respectively.

The following research hypotheses were tested: (1) shear bond strength to dentin is influenced by (i) universal adhesive type, (ii) adhesive application protocol, and (iii) dentin contamination condition; (2) 1% (w/v) gallic acid preconditioning affects residual iron content on ferric sulfate-contaminated dentin surfaces through interaction with ferric sulfate residues. The present findings are expected to carry implications for the development of biointeractive, minimally invasive dentin conditioning strategies that prioritize biological substrate integrity alongside short-term mechanical performance, an area of active clinical and materials science inquiry [8, 17].

## Materials and methods

### Experimental design

This study employed a balanced, three-factor, fully crossed experimental design. The primary outcome was shear bond strength (MPa) of the bonded composite-dentin interface. Secondary outcomes included failure mode distribution, residual iron content (EDS elemental analysis), and dentin surface morphology (SEM qualitative assessment).

The independent variables were: (1) universal adhesive type [two levels: ABU, TUB], (2) adhesive application protocol [three levels: SE, CTE, MTE], and (3) dentin contamination condition [two levels: noncontaminated, ferric sulfate [FS]-contaminated]. This design yielded 12 experimental groups (2 × 3 × 2), each comprising 15 specimens (*n* = 15) for shear bond strength testing (total *n* = 180).

Additional specimens were prepared for SEM (*n* = 3 per group; 6 groups; total *n* = 18) and EDS analysis (*n* = 5 per group; 5 groups; total *n* = 25). All restorative and bonding procedures were performed by a single, calibrated investigator. To minimize observer bias, specimens were assigned randomized numerical codes prior to testing; group allocation was concealed during SBS testing, data entry, and failure mode assessment, and was revealed only after all measurements were recorded and verified.

### Shear bond strength analysis

#### Power analysis

A priori power analysis was conducted using G*Power software (version 3.1.9.7; Universität Düsseldorf, Germany) to determine the minimum required sample size for a three-way factorial ANOVA. The experimental design included three independent variables: universal adhesive type (All Bond Universal or Tokuyama Universal Bond), dentin contamination condition (noncontaminated or ferric sulfate-contaminated), and adhesive application protocol (self-etch [SE], conventional total-etch [CTE], or modified total-etch [MTE]). Based on preliminary pilot data from the laboratory and an estimated effect size of f = 0.38, the minimum required total sample size was calculated to be 132 specimens to achieve 80% statistical power at an α level of 0.05. To ensure adequate statistical power and account for potential specimen exclusion, 180 specimens (*n* = 15 per group) were obtained from 90 extracted mandibular molars by buccolingual sectioning at the midpoint (two specimens per tooth) and employed in the present study.

Post-hoc power analysis confirmed adequate statistical power for all significant main effects: contamination condition (f = 0.305, 1-β = 0.982), adhesive type (f = 0.446, 1-β > 0.999), and application protocol (f = 0.543, 1-β > 0.999), indicating that the sample size of 180 specimens obtained from 90 mandibular molars (two specimens per tooth) was sufficient to detect the observed effects.

#### Sample preparation and group allocation

This study was approved by the Non-interventional Clinical Research Ethics Committee of Aydın Adnan Menderes University (Protocol No:160311). Ninety freshly extracted human mandibular molars were collected from patients aged 18–35 years who provided written informed consent for tooth donation for research purposes, in accordance with the Declaration of Helsinki. Teeth were extracted for orthodontic or prophylactic reasons and used within one month of extraction. Teeth were visually and radiographically inspected to exclude those with: (1) carious lesions or previous restorations, (2) structural defects (cracks, enamel fractures, hypoplastic defects), (3) significant attrition or erosion, or (4) evidence of endodontic treatment. Soft tissue remnants were removed using a periodontal curette under running water. The teeth were disinfected by immersion in a 0.1% thymol solution for 1 week and subsequently stored in distilled water at 4 °C, with the water changed daily to prevent bacterial growth.

Each tooth was sectioned buccolingually through the mesiodistal midpoint of the occlusal surface using a high-speed handpiece and diamond disc under continuous water cooling, yielding two standardized dentin specimens per tooth (mesial and distal halves). This procedure resulted in 180 specimens in total. Each specimen was individually embedded in a standardized cylindrical PVC mold, with the root in auto-polymerizing acrylic resin up to the cemento-enamel junction and the occlusal surface exposed. Each specimen was labeled with a unique identifier marked on the corresponding mold. The occlusal enamel surface of each specimen was removed with a high-speed handpiece and diamond bur under continuous water cooling to expose a flat dentin surface. To standardize the smear layer, the dentin surfaces were further abraded with 600-grit silicon carbide abrasive paper for 15 s.

Each tooth yielded two specimens: the mesial half was allocated to an ABU group and the distal half to a TUB group. Within each adhesive type, assignments for adhesive application protocol and contamination condition were determined independently using computer-generated random number sequences (https://www.randomizer.org), resulting in 12 experimental groups (*n* = 15 per group) (Table 1). Group allocation was based on three variables: universal adhesive type (ABU or TUB), adhesive application protocol (SE, CTE, or MTE), and dentin contamination condition (noncontaminated or ferric sulfate-contaminated). Table 1 summarizes the nomenclature of the experimental groups based on these three factors. Table 2 presents the composition and specifications of the materials used in the study.

**Table 1:** Experimental Group Allocation Based on Study Variables

Adhesive Type	Application Protocol	Noncontaminated	Ferric Sulfate-Contaminated
ABU	SE	ABU-SE	FS_ABU-SE
	CTE	ABU-CTE	FS_ABU-CTE
	MTE	ABU-MTE	FS_ABU-MTE
TUB	SE	TUB-SE	FS_TUB-SE
	CTE	TUB-CTE	FS_TUB-CTE
	MTE	TUB-MTE	FS_TUB-MTE

*ABU* All Bond Universal, *TUB* Tokuyama Universal Bond, *SE* Self-Etch, *CTE* Conventional Total-Etch with 35% phosphoric acid, *MTE* Modified Total-Etch with 1% (w/v) gallic acid, *FS* Ferric Sulfate

**Table 2:** Materials Used in the Study

Material	Manufacturer	Composition/Specifications	Application Mode
All Bond Universal (ABU)	Bisco Inc., Schaumburg, IL, USA	Bis-GMA, 10-MDP, HEMA, ethanol, water, photoinitiators, pH = 3.2 Lot: 2,000,002,692	**SE**: two coats, scrubbing motion (10–15 s/coat), air-dried (≥ 10 s), light-cured (10 s, 1000 mW/cm²) **CTE**: preceded by 35% H₃PO₄ (10 s, rinsed 10 s), then SE protocol as above **MTE**: preceded by 1% gallic acid (10 s, rinsed 10 s), then SE protocol as above
Tokuyama Universal Bond* (TUB)	Tokuyama Dental Corp., Tokyo, Japan	Bond A: Phosphoric acid monomer (3D-SR monomer), HEMA, TEG-DMA, bis-GMA, MTU-6, acetone Bond B: acetone, isopropyl alcohol, water, acryl borate catalyst, γ-MPTES, peroxide; pH = 2.2 Lot: #005	**SE**: Bond A + B mixed (1:1), scrubbing motion (10 s), air-dried (~ 30 s), self-cured **CTE**: preceded by 35% H₃PO₄ (10 s, rinsed 10 s), then SE protocol as above **MTE**: preceded by 1% gallic acid (10 s, rinsed 10 s), then SE protocol as above
Filtek Z250	Solventum, St. Paul, MN, USA	Bis-GMA, UDMA, Bis-EMA, TEGDMA, Silane-treated ceramic, Aluminum oxide, Lot: N880786	Single increment (2 mm height, 4 mm diameter); light-cured (20 s, 1000 mW/cm²)
K-Etchant	Kuraray Noritake Dental, Tokyo, Japan	35% phosphoric acid, colloidal silica, water, pigment, pH = 1.9 Lot: 2Q0035	Applied for10 s; rinsed 10 s; air-dried
ViscoStat	Ultradent Products Inc., South Jordan, UT, USA	20% ferric sulfate (FS), pH ~ 1.0 Lot: C15CN	Applied for 3 min; rinsed 3 min
Gallic acid	Sigma-Aldrich Chemie, Steinheim, Germany	≥ 97.5% purity, 3,4,5-trihydroxybenzoic acid, (C_7_H_6_O_5_), pH = 2.8 (pH value of the 1% (w/v) gallic acid solution) Lot: SLBV4564.	Applied with a microbrush, scrubbing motion (10 s); rinsed 10 s

* Original formulation (not TokuyamaUniversal Bond II); contains 3D-SR monomer without 10-MDP

*3D-SR* three dimensional self-reinforcing, *10-MDP* methacryloyloxydecyl dihydrogen phosphate, *HEMA* 2-hydroxyethyl methacrylate, *MTU-6* 6-methacryloyloxyhexyl 2-thiouracil-5-carboxylate, *TEG-DMA* triethylene glycol dimethacrylate, *γ* *MPTES* 3-methacryloxypropyl trimethoxy silane, *UDMA* urethane dimethacrylate, *bis-EMA* bisphenol A ethoxylate dimethacrylate. *ABU* All Bond Universal, *TUB* Tokuyama Universal Bond, *SE* self-etch, *TE* total-etch, *MTE* modified total-etch

#### Restorative procedures

##### Dentin surface contamination protocol

Dentin specimens were divided into two contamination conditions:

Noncontaminated groups (ABU-SE, ABU-CTE, ABU-MTE, TUB-SE, TUB-CTE, TUB-MTE): No contamination was applied prior to the application of the adhesive.

Ferric sulfate-contaminated groups (FS_ABU-SE, FS_ABU-CTE, FS_ABU-MTE, FS_TUB-SE, FS_TUB-CTE, FS_TUB-MTE): ViscoStat hemostatic gel (20% ferric sulfate, Ultradent Products Inc., South Jordan, UT, USA; pH ~ 1.0) was applied to dentin surfaces for 3 min according to manufacturer instructions, then rinsed thoroughly with distilled water for 3 min.

##### Universal adhesive application protocols

Two universal adhesives, All Bond Universal and Tokuyama Universal Bond, were applied using three protocols:

##### Self-etch (SE) protocol

Universal adhesives were applied directly to dentin without preconditioning. All Bond Universal was dispensed and applied in two consecutive coats using a scrubbing motion (10–15 s per coat) according to the manufacturer’s instructions. Excess solvent was evaporated using oil-free air pressure (held ~ 10 cm from the surface) for at least 10 s until adhesive movement ceased. The adhesive was then light-cured for 10 s at 1000 mW/cm² using an LED curing unit (BlueLEX LD-109, Monitex, New Taipei City, Taiwan), with light output verified using a radiometer (Peng Lim Enterprise, Kaohsiung, Taiwan).

Tokuyama Universal Bond used in this study was the original formulation (Tokuyama Dental Corp., Tokyo, Japan), a two-component self-curing universal adhesive (3D-SR monomer; no 10-MDP), as described in the Introduction. Bond A and Bond B (one drop each) were dispensed and mixed according to the manufacturer’s specifications until a uniform green color was achieved. The adhesive was applied with a scrubbing motion for 10 s, followed by continuous air-drying (using oil-free air pressure) until solvent evaporation was complete and the adhesive ceased flowing (~ 30 s total). As this adhesive employs a self-cure mechanism, light curing was omitted. This protocol was used for ABU-SE, TUB-SE, FS_ABU-SE, and FS_TUB-SE groups.

##### Conventional total-etch (CTE) protocol

Dentin was preconditioned with 35% phosphoric acid gel (K-Etchant Syringe, Kuraray Noritake Dental, Tokyo, Japan; pH 1.9) for 10 s, rinsed with distilled water for 10 s, gently air-dried, and then the corresponding adhesive was applied as described above. This protocol was used for ABU-CTE, TUB-CTE, FS_ABU-CTE, and FS_TUB-CTE groups.

##### Modified total-etch (MTE) protocol

An experimental 1% (w/v) aqueous gallic acid solution was prepared by dissolving 1 g of gallic acid powder (Sigma-Aldrich Chemie, Steinheim, Germany) in 100 mL of distilled water. The gallic acid solution was freshly prepared before each experimental session, protected from light, and used within 24 h of preparation. pH stability was verified immediately before application using a calibrated pH meter (Hanna, pH211, Rhode Island, USA), confirming pH 2.8. Dentin was preconditioned with this solution using a microbrush in a scrubbing motion for 10 s, then rinsed thoroughly with distilled water for 10 s, and then the corresponding adhesive was applied as described above. Since gallic acid is not commercially available as a dental material and lacks manufacturer-provided guidelines, the protocol was adapted from that of phosphoric acid conditioning. The 1% (w/v) concentration and 10-second application time were selected to maintain consistency with the protocol established in our prior work [17] and to enable direct comparison with the phosphoric acid conditioning step, which was applied for the same duration according to the manufacturer’s instructions.

This protocol was used for ABU-MTE, TUB-MTE, FS_ABU-MTE, and FS_TUB-MTE groups.

##### Composite resin restorations and artificial aging

Composite resin build-ups (2 mm height, 4 mm diameter) were fabricated using transparent plastic molds filled with microhybrid composite resin (Filtek Z250, Solventum, St. Paul, MN, USA; shade A2) in a single increment and light-cured for 20 s at 1000 mW/cm² using the same LED unit employed for adhesive polymerization. After light-curing, the plastic molds were carefully removed with a scalpel. Specimens were stored in distilled water at 37 °C for 24 h in an incubator and then subjected to thermocycling for 5,000 cycles between 5 °C and 55 °C (with a 30-second dwell time and a 10-second transfer time), which has been proposed to approximate 6 months of intraoral aging [40, 41], although the clinical equivalence of thermocycling protocols remains a subject of debate.

#### Shear bond strength testing

Following thermocycling, shear bond strength (SBS) analysis was performed using a universal testing machine (Shimadzu, Tokyo, Japan) at a crosshead speed of 0.5 mm/min. Shear bond strength testing was selected to enable direct comparison with prior studies employing the same geometry [17] and to be consistent with the established methodology in the contamination literature [11–14, 17]. Microshear bond strength testing was not employed, as the bonded area geometry (4 mm-diameter composite cylinder) was specifically selected to replicate the methodology of the prior study [17], enabling direct inter-study comparison and facilitating contextual interpretation within this literature base.

A chisel-shaped loading device was positioned parallel to and at the composite-dentin interface. The bonding surface area was fixed at 12.56 mm² (corresponding to a circular bonded area with 4 mm diameter, πr²), and SBS was calculated by dividing the maximum force (N) at failure by the bonded area, expressed in MPa. Shear bond strength testing was performed by the sole investigator. To minimize observer bias, each specimen was assigned a randomized numerical code prior to testing; the investigator remained unaware of group identities during testing and data entry, with group allocation revealed only after all SBS values were recorded and verified for accuracy.

#### Failure mode assessment

Following shear bond strength testing, all fractured specimens were examined under a stereomicroscope (Olympus SZ61, Munster, Germany) at 40× magnification. Failure modes were classified according to established criteria [7]:

- Adhesive failure: Complete interfacial debonding between adhesive resin and dentin substrate, with no visible dentin or composite remnants on the opposing surface.
- Mixed failure: Combined interfacial failure with partial substrate involvement, characterized by regions of exposed dentin alongside areas with residual adhesive or composite.
- Cohesive failure in dentin: Fracture occurring entirely within the dentin substrate.
- Cohesive failure in composite: Fracture occurring entirely within the composite resin.

Failure mode assessment was performed by a single calibrated examiner who was blinded to group allocation using the same coding system described above. Representative digital images of each failure type were captured using a camera system (Olympus cellSens Standard, Munster, Germany) attached to the stereomicroscope. The distribution of failure modes was recorded as frequency counts and percentages for descriptive characterization of bonding behavior across experimental conditions.

### Scanning electron microscopy (SEM) analysis

#### Sample preparation for SEM

Eighteen dentin discs were prepared from additional extracted mandibular molars. The apical portion of each root was embedded in auto-polymerizing acrylic resin, with the acrylic level set approximately 2–3 mm apical to the cemento-enamel junction, leaving the coronal root segment and crown exposed to facilitate handling and disc sectioning. The occlusal enamel surface was removed with a high-speed handpiece and diamond bur under continuous water cooling to expose a flat dentin surface. A 2 mm-thick dentin disc was then obtained by sectioning from the apical aspect using a high-speed handpiece and diamond disc under continuous water cooling. To standardize the smear layer, the exposed occlusal dentin surface of each disc was wet-ground with 600-grit SiC abrasive paper for 15 s. Specimens were randomly assigned to six experimental groups (*n* = 3 per group):

1. Control: Standardized smear layer without additional treatment.
2. CTE: 35% phosphoric acid conditioning (10 s).
3. MTE: 1% (w/v) gallic acid conditioning (10 s).
4. FS_SE: Ferric sulfate contamination (ViscoStat application) (3 min) only.
5. FS_CTE: Ferric sulfate contamination (ViscoStat application) followed by 35% phosphoric acid conditioning.
6. FS_MTE: Ferric sulfate contamination (ViscoStat application) followed by 1% (w/v) gallic acid conditioning.

All treatment protocols were identical to those described in Section Restorative procedures. After surface treatment, dentin disc specimens were ultrasonically cleaned in deionized water for 15 min at 40 kHz, then dried in a desiccator for 12 h. They were sputter-coated with gold and stored in a desiccator until imaging. A minimum of three imaging locations per specimen were examined to enhance the representativeness of morphological observations; however, given the small sample size (*n* = 3 per group), the selected micrographs should be regarded as illustrative of predominant surface features rather than statistically representative of the full morphological range within each group. To minimize potential observer bias, representative micrographs were independently reviewed by a second examiner, and consensus was reached on all morphological descriptions.

#### SEM imaging and qualitative assessment

Representative images were captured at ×25,000 magnification, with at least 3 different locations examined per specimen. All imaging was performed under high-vacuum mode using an Everhart-Thornley detector (ETD) at an accelerating voltage of 3.00 kV and a spot size of 3.0. Representative micrographs of the Control, CTE, MTE, FS_SE, FS_CTE, and FS_MTE groups are presented in Figs. 2, 3, 4, 5, 6 and 7, respectively. Morphological assessment was entirely qualitative and descriptive in nature. No quantitative morphometric measurements were performed; all SEM interpretations were based exclusively on qualitative visual assessment of the micrographs, focusing on apparent smear layer continuity and removal, dentin tubule visibility and orifice morphology, peritubular and intertubular dentin surface features, visually identifiable surface deposits, and overall surface architecture.

### Energy-dispersive X-ray spectroscopy (EDS) analysis

#### Sample preparation for EDS

Twenty-five dentin discs were prepared following the same protocol described in Section Sample preparation for SEM. Each dentin surface was wet-ground with 600-grit SiC paper for 15 s to produce a standardized smear layer. Based on the dentin surface pretreatment protocol, an unconditioned control group was not included in the EDS analysis, as the primary objective was to compare the elemental profiles of conditioning protocols rather than to establish an absolute compositional baseline. Specimens were randomly assigned to five experimental groups (*n* = 5 per group):

1. CTE: Dentin surface received 35% phosphoric acid conditioning.
2. MTE: Dentin surface received 1% (w/v) gallic acid conditioning.
3. FS_SE: Dentin surface received ferric sulfate contamination (ViscoStat application) only (self-etch simulation).
4. FS_CTE: Dentin surface received ferric sulfate contamination (ViscoStat application) followed by 35% phosphoric acid conditioning.
5. FS_MTE: Dentin surface received ferric sulfate contamination (ViscoStat application) followed by 1% (w/v) gallic acid conditioning.

#### EDS measurements and data collection

After pretreatment, all specimens were cleaned ultrasonically in deionized water for 15 min and then dried in a desiccator for 12 h. To detect residual iron after application of the 20% ferric sulfate-containing hemostatic agent and subsequent conditioning protocols, elemental analysis was performed using an energy-dispersive X-ray spectroscopy system (Oxford Instruments Aztec, Abingdon, UK) integrated with the scanning electron microscope.

Analysis parameters were as follows: accelerating voltage, 15.0 kV; spot size, 5.0; working distance, 10.6 mm; and acquisition time, 60 s per specimen. Spectra were obtained using whole-surface scans encompassing the entire prepared dentin area, thereby providing an average elemental composition of the treated surface rather than localized point measurements. A minimum count rate of 2000 counts per second was used to ensure adequate signal quality. Elements with atomic percentages below 0.01% were considered below the detection limit of the system.

The analysis was designed to detect residual iron derived from ferric sulfate contamination and to compare iron retention among conditioning protocols. In addition, the elemental composition of the treated dentin surfaces, including carbon, nitrogen, oxygen, sodium, magnesium, aluminum, phosphorus, and calcium, was recorded. The calcium-to-phosphorus atomic ratio was calculated for each specimen as a complementary indicator of relative mineral preservation across treatment groups.

### Statistical analysis

Quantitative data from shear bond strength testing were analyzed using IBM SPSS Statistics (Version 23.0; IBM Corp., Armonk, NY, USA). Data normality was assessed using the Shapiro-Wilk test, and homogeneity of variances was verified with Levene’s test. A three-way analysis of variance (ANOVA) was performed to evaluate the main effects of dentin contamination condition, universal adhesive type, and adhesive application protocol, as well as all two-way and three-way interaction terms.

Post-hoc pairwise comparisons for significant main effects (α = 0.05) were performed using Tukey’s Honest Significant Difference (HSD) test, which controls the family-wise error rate across multiple comparisons. For factors with only two levels (e.g., contamination condition, adhesive type), the main effect F-test provides the pairwise comparisons. Effect sizes for significant effects were reported as partial eta-squared (ηp²), with values of 0.01, 0.06, and 0.14 interpreted as small, medium, and large effects, respectively, according to Cohen’s conventions. Results are reported as mean ± standard deviation (SD), and statistical significance was set at α = 0.05.

The statistical unit for shear bond strength analysis was the individual bonded specimen. Each tooth contributed two specimens: the mesial half was assigned to an ABU group and the distal half to a TUB group, with protocol and contamination condition randomized independently for each half within its respective adhesive type. This split-tooth allocation functioned as a within-tooth matched design for the adhesive type comparison, controlling for tooth-level variation in dentin quality and tubule morphology, a methodological advantage over fully randomized allocation. Although this design introduces within-tooth correlation for the adhesive type factor, the two specimens from each tooth were assigned to different adhesive types; consequently, any residual within-tooth correlation would be expected to act conservatively on the adhesive type comparison, reducing rather than inflating the F-statistic, and is therefore unlikely to account for the observed highly significant effect (F = 33.345, *p* < 0.001). The tooth was not modeled as a random effect, consistent with standard practice in dental bonding research; this limitation is acknowledged.

For EDS analysis, descriptive statistics (mean ± standard deviation, *n* = 5 per group) were calculated to characterize the elemental composition profiles across treatment groups. The primary outcome for testing the second research hypothesis was the presence or absence of detectable iron on conditioned dentin surfaces. Iron detection was considered positive when the atomic percentage exceeded the instrument’s detection limit (0.01 atomic%), and negative when it was below this threshold. Within each group, all five specimens yielded consistent results: either iron was detectable in every specimen or absent in every specimen, leaving no ambiguity requiring statistical testing. The numerical proximity of FS_SE and FS_MTE iron values (0.57 ± 0.04 vs. 0.62 ± 0.13 atomic%) is noted; however, quantitative comparison between contaminated groups was not the focus of the first hypothesis and is therefore reported descriptively.

For SEM analysis, a qualitative morphological assessment (*n* = 3 per group) was performed to characterize surface features and corroborate findings from quantitative analyses. The small sample size reflected the descriptive, non-hypothesis-testing nature of this component, which provided visual documentation of surface treatments rather than statistical inference.

## Results

### Shear bond strength results

Before conducting the three-way ANOVA, the assumptions for parametric analysis were evaluated. Homogeneity of variances was confirmed by Levene’s test (F(11, 168) = 1.305, *p* = 0.225). Data normality was confirmed for all groups by the Shapiro-Wilk test (all *p* > 0.05), validating the use of parametric analysis. The relatively low between-specimen variability observed in some groups likely reflects the highly standardized surface preparation protocol (single-session 600-grit SiC abrasion by a single investigator), which minimizes surface heterogeneity compared with less controlled procedures. Individual SBS values for all 180 specimens are presented graphically in Supplementary Figure S1.

Three-way ANOVA revealed statistically significant main effects for all three independent variables:

- Contamination condition: F(1, 168) = 15.612, *p* < 0.001, ηp²=0.085.
- Adhesive type: F(1, 168) = 33.345, *p* < 0.001, ηp²=0.166.
- Adhesive application protocol: F(2, 168) = 24.761, *p* < 0.001, ηp²=0.228.

No statistically significant two-way or three-way interaction effects were observed (all *p* > 0.05), indicating that the influence of each independent variable on bond strength was additive rather than synergistic (Table 3). The model explained 38.4% of the variance in shear bond strength (R² = 0.384, adjusted R² = 0.344).

**Table 3:** Three-Way ANOVA Results for Shear Bond Strength

Source	SS	df	MS	F	*p*	ηp²
Contamination	122.694	1	122.694	15.612	**< 0.001**	0.085
Adhesive Type	262.064	1	262.064	33.345	**< 0.001**	0.166
Protocol	389.201	2	194.601	24.761	**< 0.001**	0.228
Contamination × Adhesive	0.009	1	0.009	0.001	0.973	0.000
Contamination × Protocol	17.862	2	8.931	1.136	0.323	0.013
Adhesive × Protocol	0.592	2	0.296	0.038	0.963	0.000
Contamination × Adhesive × Protocol	31.147	2	15.574	1.982	0.141	0.023

*SS* Sum of Squares, *df* degrees of freedom, *MS* Mean Square, *ηp²* partial eta-squared. R² = 0.384, Adjusted R² = 0.344. Error df = 168

Contamination condition significantly affected bond strength (*p* < 0.001). Noncontaminated dentin specimens exhibited higher mean bond strength (20.35 ± 3.44 MPa) compared to those contaminated with ViscoStat (18.69 ± 3.30 MPa), indicating compromised bonding performance under contaminated conditions (Table 4).

**Table 4:** Mean Shear Bond Strength (MPa) by Adhesive Type, Application Protocol, and Dentin Contamination Condition

Adhesive Type	Application Protocol	Noncontaminated	Ferric Sulfate-Contaminated	Total
ABU	SE	19.31 ± 2.42	17.89 ± 2.13	18.60 ± 2.35
	CTE	22.55 ± 3.44	20.61 ± 1.66	21.58 ± 2.83
	MTE	22.82 ± 2.87	21.18 ± 2.62	22.00 ± 2.82
	**Total**	21.56 ± 3.30	19.89 ± 2.57	20.73 ± 3.06
TUB	SE	18.14 ± 2.91	14.50 ± 3.20	16.32 ± 3.54
	CTE	19.13 ± 3.28	18.91 ± 2.97	19.02 ± 3.07
	MTE	20.12 ± 3.21	19.08 ± 2.37	19.60 ± 2.82
	**Total**	19.13 ± 3.17	17.50 ± 3.53	18.31 ± 3.44
Total	SE	18.73 ± 2.70	16.20 ± 3.18	17.46 ± 3.19 ^**a**^
	CTE	20.84 ± 3.73	19.76 ± 2.52	20.30 ± 3.20 ^**b**^
	MTE	21.47 ± 3.29	20.13 ± 2.68	20.80 ± 3.05 ^**b**^
	**Total**	20.35 ± 3.44	18.69 ± 3.30	19.52 ± 3.46

^**a, b**^ Different superscript letters indicate statistically significant differences between protocols (Tukey’s HSD, *p* < 0.05). Values are presented as mean ± standard deviation (SD). ABU: All Bond Universal; TUB: Tokuyama Universal Bond; SE: Self-Etch; CTE: Conventional Total-Etch; MTE: Modified Total-Etch

Universal adhesive type also demonstrated a significant effect (*p* < 0.001). Specimens bonded with All Bond Universal exhibited higher mean bond strengths (20.73 ± 3.05 MPa) than those bonded with Tokuyama Universal Bond (18.31 ± 3.44 MPa), demonstrating the statistically higher overall mean bond strength of ABU across all tested conditions (Table 4).

Adhesive application protocol significantly influenced bond strength (*p* < 0.001). The self-etch protocol yielded the lowest mean bond strength (17.46 ± 3.19 MPa), whereas both conventional total-etch and modified total-etch protocols demonstrated significantly higher values (20.30 ± 3.20 MPa and 20.80 ± 3.05 MPa, respectively) (Table 4).

Post-hoc pairwise comparisons (Tukey’s HSD) revealed that the SE protocol yielded significantly lower bond strengths compared to both CTE (mean difference = 2.84 MPa, 95% CI [1.63, 4.05], *p* < 0.001) and MTE protocols (mean difference = 3.34 MPa, 95% CI [2.13, 4.55], *p* < 0.001). However, no significant difference was detected between the CTE and MTE protocols (mean difference = 0.50 MPa, *p* = 0.591), indicating comparable bonding efficacy for phosphoric acid and gallic acid preconditioning under the tested conditions.

According to partial eta squared values, the adhesive application protocol exerted the greatest influence on shear bond strength (ηp²=0.228, large effect), followed by adhesive type (ηp²=0.166, large effect) and contamination condition (ηp²=0.085, medium effect).

### Failure mode analysis

**Fig. 1:**
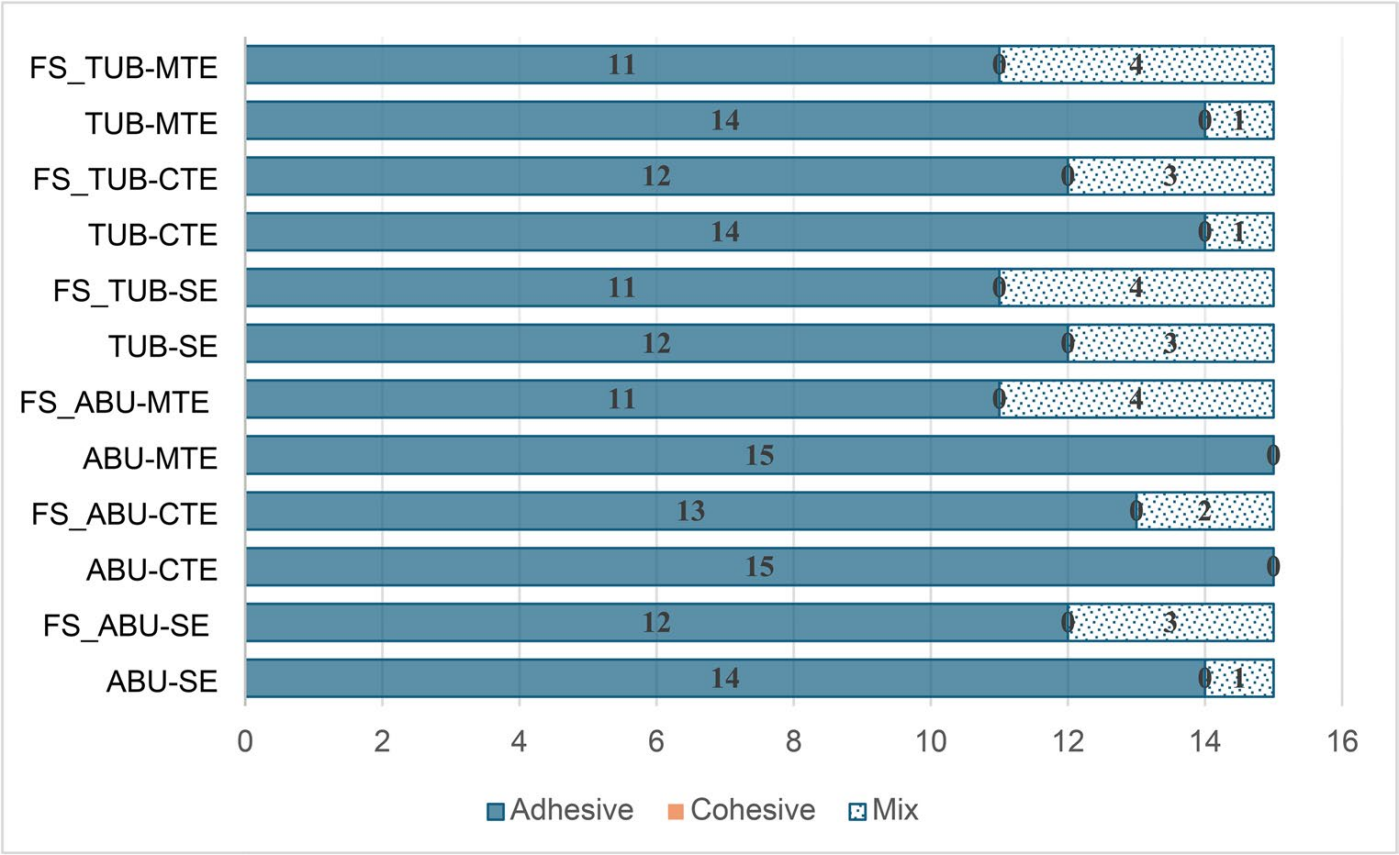
Distribution of fracture modes across all experimental groups. Adhesive, cohesive, and mixed failure modes are presented as specimen counts; no cohesive failures in dentin or composite were observed in any group. Numbers within bars indicate specimen counts; corresponding percentages for each group are reported in the Results section. ABU: All Bond Universal; TUB: Tokuyama Universal Bond; SE: Self-Etch; CTE: Conventional Total-Etch; MTE: Modified Total-Etch; FS: Ferric Sulfate

Post-fracture examination revealed adhesive or mixed failures in all specimens, with no cohesive failures observed in either the dentin or composite substrates (Fig. 1).

All Bond Universal: Under noncontaminated conditions, the ABU-SE group showed minimal mixed failures (6.7%), while the ABU-CTE and ABU-MTE groups achieved 100% adhesive failures (15/15 each). ViscoStat contamination increased mixed failures across all protocols, most notably in gallic acid-treated specimens (FS_ABU-MTE: 26.7%) compared to phosphoric acid-treated specimens (FS_ABU-CTE: 13.3%) and self-etch groups (FS_ABU-SE: 20.0%).

Tokuyama Universal Bond: All protocols exhibited some mixed failures even under noncontaminated conditions (6.7–20.0%). Contamination further increased the proportion of mixed failures, with FS_TUB-SE (26.7%), FS_TUB-CTE (20.0%), and FS_TUB-MTE (26.7%) showing elevated mixed-failure rates.

Across both adhesive systems, adhesive failures predominated (73.3–100%), and ferric sulfate contamination consistently elevated mixed failure proportions, most markedly in gallic acid-preconditioned groups (26.7%).

### SEM results

**Fig. 2:**
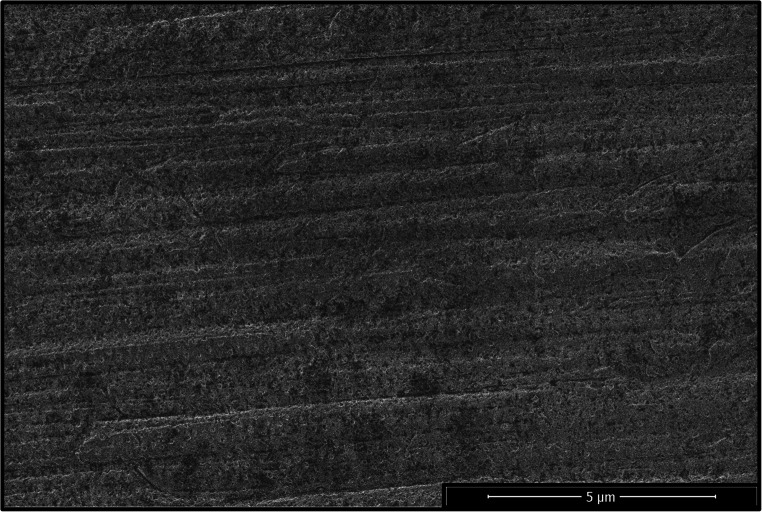
Representative scanning electron micrograph of control dentin surface after standardized 600-grit SiC abrasion. Complete dentin surface coverage with continuous, homogeneous smear layer (*n* = 3). No tubule orifices were discernible, confirming complete continuity of the smear layer. High-vacuum mode; ETD detector; 3.00 kV; ×25,000; scale bar = 5 μm

**Fig. 3:**
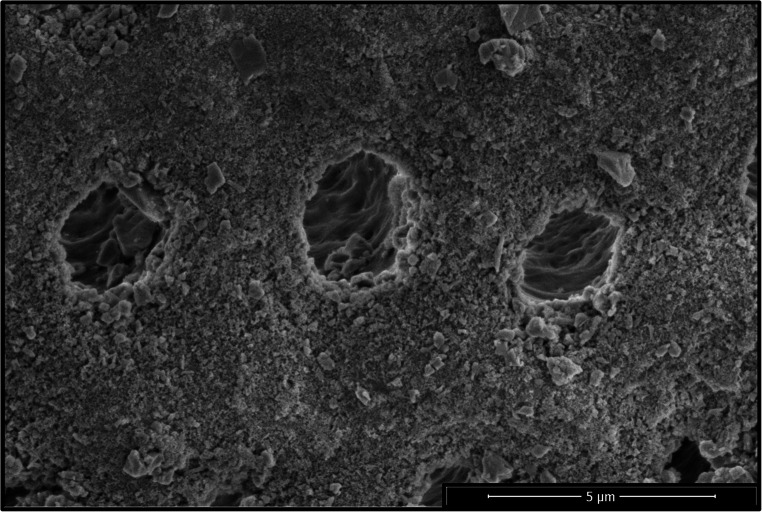
Representative scanning electron micrograph of dentin treated with conventional total-etch (CTE) protocol. After 35% phosphoric acid conditioning (10 s, *n* = 3), complete smear layer removal with widely opened tubule orifices and a rough, granular, porous intertubular surface texture. Peritubular dentin was discernible as subtle raised borders surrounding each orifice. High-vacuum mode; ETD detector; 3.00 kV; ×25,000; scale bar = 5 μm

**Fig. 4:**
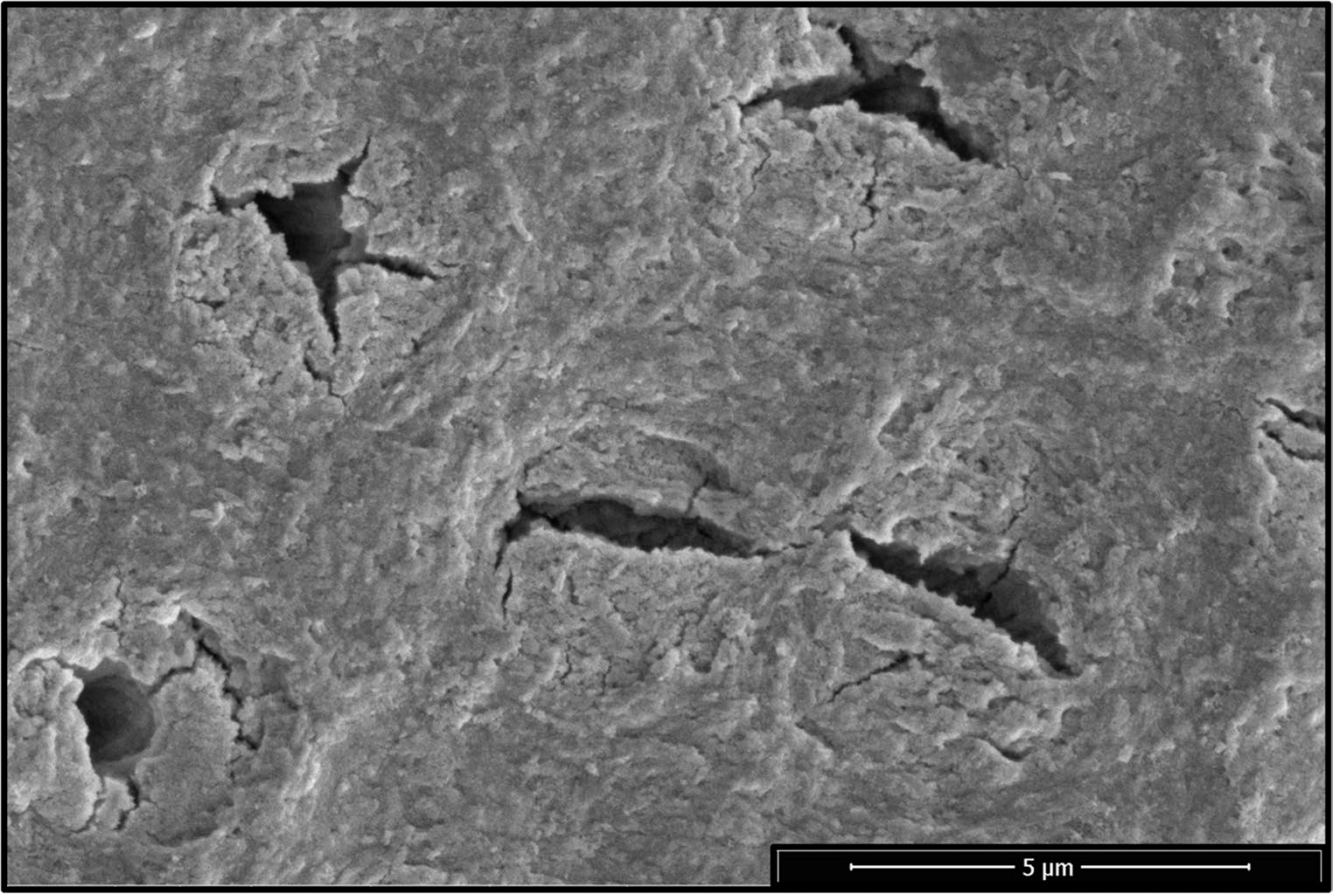
Representative scanning electron micrograph of dentin treated with modified total-etch (MTE) protocol. After 1% (w/v) gallic acid conditioning (10 s, *n* = 3), partially open tubule orifices with irregular, elongated contours. The intertubular surface retained a discontinuous residual coating exhibiting fine fissuring, with no discrete surface deposits. Surface morphology intermediate between the Control and CTE groups. High-vacuum mode; ETD detector; 3.00 kV; ×25,000; scale bar = 5 μm

**Fig. 5:**
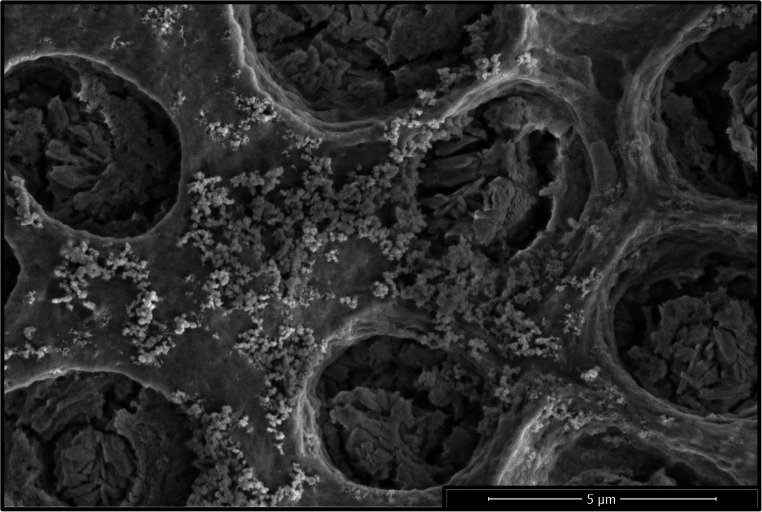
Representative scanning electron micrograph of FS-Contaminated dentin without subsequent conditioning (FS_SE) protocol. After ferric sulfate contamination (ViscoStat application, 3 min, *n* = 3), coarse granular-to-crystalline precipitate clusters were distributed across the intertubular dentin surface and around tubule peripheries. Deposits were also visible within tubule lumens. Tubule orifices remained patent despite the overlying precipitates. High-vacuum mode; ETD detector; 3.00 kV; ×25,000; scale bar = 5 μm

**Fig. 6:**
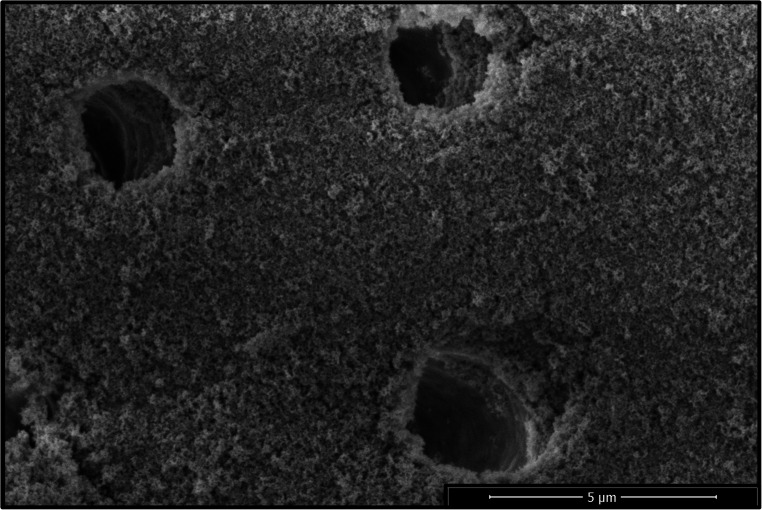
Representative scanning electron micrograph of FS-Contaminated dentin treated with conventional total-etch (FS_CTE) protocol. Ferric sulfate contamination, followed by 35% phosphoric acid conditioning (10 s, *n* = 3). No visually identifiable ferric sulfate-associated surface deposits. Tubule orifices were identifiable across the surface. Surface morphology closely resembled that of noncontaminated CTE specimens. No residual granular precipitates or aggregate deposits were identified by SEM morphological assessment. High-vacuum mode; ETD detector; 3.00 kV; ×25,000; scale bar = 5 μm

**Fig. 7:**
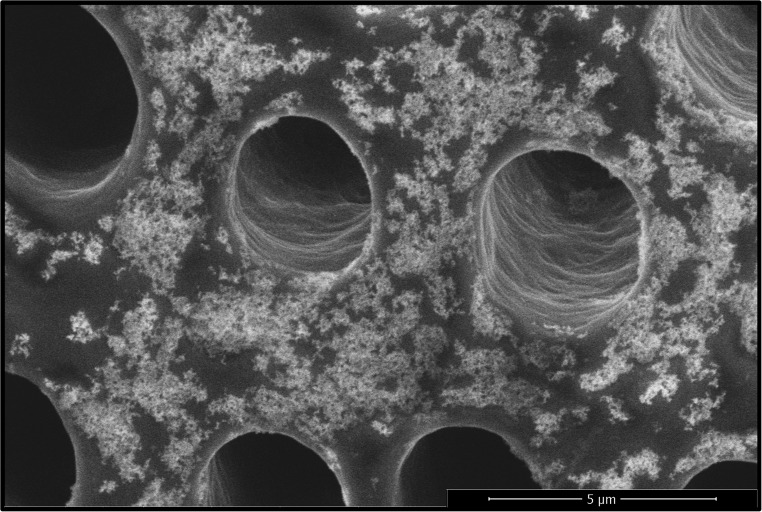
Representative scanning electron micrograph of FS-Contaminated dentin treated with modified total-etch (FS_MTE). Ferric sulfate contamination followed by 1% (w/v) gallic acid conditioning (10 s, *n* = 3). Partial decontamination with residual granular deposits visible on intertubular dentin. Heterogeneous tubule opening, incomplete iron removal. High-vacuum mode; ETD detector; 3.00 kV; ×25,000; scale bar = 5 μm

Representative scanning electron micrographs of dentin surfaces following different conditioning protocols are presented in Figs. 2, 3, 4, 5, 6 and 7. Qualitative morphological assessment revealed distinct surface characteristics across experimental groups, reflecting differences in demineralization capacity and the conditioning agents’ surface-modification effects.

#### Control group

The control dentin surface exhibited a continuous, homogeneous smear layer with an amorphous, granular texture, completely obscuring the underlying dentinal tubules (Fig. 2). Parallel linear abrasion scratches from standardized 600-grit SiC polishing were prominently visible across the entire surface, imparting a directional texture consistent with a mechanically produced smear layer. No tubule orifices were discernible, confirming complete smear-layer continuity and establishing this as the standardized baseline substrate condition.

#### Conventional total-etch (CTE) group

Phosphoric acid conditioning resulted in the complete removal of the smear layer and extensive demineralization of the dentin surface (Fig. 3). Dentin tubules were widely opened. Peritubular dentin was discernible as a subtle raised border surrounding each orifice. The intertubular dentin surface exhibited a rough, granular, and porous texture. Scattered fine mineral debris was focally noted on the demineralized surface; however, no coherent smear layer remnants or aggregate surface precipitates were identified.

#### Modified total-etch (MTE) group

Gallic acid conditioning produced a distinctly milder, more conservative surface modification than phosphoric acid (Fig. 4). Tubule orifices were visible but appeared narrower and more irregularly configured than those in CTE specimens, with many exhibiting an elongated or slit-like morphology. Peritubular dentin rims were indistinct, appearing only as subtle topographic elevations. The intertubular dentin surface retained a partially continuous surface coating, appearing as a discontinuous film exhibiting fine fissuring and localized disruption. No discrete surface precipitates or deposit clusters were observed. Surface morphology was intermediate between the Control (no visible tubule openings, intact smear layer) and the CTE (extensive tubule openings, complete smear layer removal) groups.

#### Ferric sulfate-self-etch (FS_SE) group

Ferric sulfate contamination (application of ViscoStat hemostatic agent) without subsequent conditioning resulted in widespread deposition of characteristic granular and crystalline precipitates across the dentin surface (Fig. 5). Dentinal tubule orifices remained visible and patent across the surface. Coarse granular to crystalline precipitate clusters were distributed across the intertubular dentin surface and around tubule peripheries. Deposits were also visible within tubule lumens, appearing as irregularly shaped, block-like aggregates occupying portions of the tubule interior. The overall surface architecture appeared rougher and more topographically irregular than that of control specimens, with deposit aggregates forming discrete, bright clusters distributed throughout the observable field.

#### Ferric sulfate-conventional total-etch (FS_CTE) group

Phosphoric acid conditioning following ferric sulfate (ViscoStat) contamination resulted in the absence of visually identifiable ferric sulfate-associated surface deposits and removal of the smear layer (Fig. 6). No residual granular precipitates, aggregate deposits, or obscured tubule orifices attributable to ferric sulfate contamination were identified, a morphological pattern consistent with the iron levels below the EDS detection limit reported for the FS_CTE group. Tubule orifices were identifiable across the surface. The resulting surface morphology closely resembled that of noncontaminated CTE specimens (Fig. 3). Peritubular dentin was discernible as a subtle raised border surrounding each orifice. The intertubular dentin surface exhibited a rough, granular, and porous texture.

#### Ferric sulfate-modified total-etch (FS_MTE) group

Gallic acid conditioning following ferric sulfate (ViscoStat) contamination resulted in partial surface decontamination with a distinctly characteristic deposit morphology (Fig. 7). Dentinal tubule orifices were patent and visible across the surface. Abundant fine, loosely aggregated precipitates were distributed extensively across the intertubular dentin surface and around tubule peripheries, covering a substantial proportion of the visible surface area. The morphological character of these deposits differed from the coarser, block-like crystalline aggregates observed in FS_SE specimens (Fig. 5), appearing finer and more broadly dispersed across the surface. No coherent smear layer continuity was observed.

#### Comparative morphological summary

A qualitative comparison of the six experimental groups revealed distinct surface morphologies. Control specimens displayed intact smear layers with completely occluded tubules. CTE specimens exhibited complete smear layer removal, with widely open tubule orifices and a rough, granular intertubular surface texture. MTE specimens showed a milder surface modification, with narrower, more irregularly configured tubule orifices and a partially intact, fissured surface coating, without the surface roughness characteristic of phosphoric acid etching. FS_SE specimens demonstrated visible tubule orifices alongside coarse granular-to-crystalline precipitate clusters distributed on the intertubular surface and within tubule lumens. FS_CTE specimens showed no visually identifiable ferric sulfate-associated surface deposits, with a surface morphology closely resembling that of noncontaminated CTE specimens. FS_MTE specimens exhibited patent tubule orifices and abundant fine, loosely aggregated precipitates distributed extensively across the intertubular dentin surface, morphologically distinct from the coarser crystalline deposits observed in FS_SE specimens. These morphological observations, particularly the presence of residual fine precipitates in FS_MTE yet their absence in FS_CTE, are consistent with the differential iron retention patterns identified by EDS analysis.

### EDS results

Elemental composition analysis by EDS revealed distinct compositional profiles across experimental groups (Table 5). Results are presented as descriptive statistics (mean ± SD, *n* = 5 per group) to characterize the elemental signatures associated with different surface treatment protocols. No inferential statistical analysis was performed on any EDS-derived data, and all findings should be interpreted accordingly.

**Table 5:** Elemental Composition of Dentin Surfaces Following Different Surface Treatment Protocols (mean ± SD, *n* = 5 per group)

Element	CTE	MTE	FS_SE	FS_CTE	FS_MTE
C	49.68 ± 0.93	29.29 ± 3.87	32.55 ± 0.29	48.37 ± 0.72	37.38 ± 3.83
N	20.61 ± 0.28	11.30 ± 1.74	14.68 ± 0.78	20.22 ± 0.33	15.22 ± 0.54
O	27.36 ± 0.48	45.03 ± 3.59	41.27 ± 0.69	28.04 ± 0.21	36.93 ± 4.22
Na	0.03 ± 0.02	0.31 ± 0.04	0.34 ± 0.01	0.06 ± 0.00	0.16 ± 0.05
Mg	0.05 ± 0.02	0.33 ± 0.11	0.28 ± 0.02	0.08 ± 0.01	0.25 ± 0.02
P	0.77 ± 0.27	5.36 ± 0.81	4.14 ± 0.15	1.15 ± 0.07	3.75 ± 0.14
Ca	1.13 ± 0.37	8.39 ± 1.14	6.18 ± 0.24	1.65 ± 0.11	5.60 ± 0.18
Fe *	< 0.01	< 0.01	**0.57 ± 0.04**	< 0.01	**0.62 ± 0.13**
Ca/P	1.47 ± 0.02	1.57 ± 0.07	1.49 ± 0.03	1.43 ± 0.02	1.49 ± 0.06

Values represent atomic percentages (mean ± SD). <0.01: Below the detection limit of the EDS system

* Fe values in bold indicate detectable iron above the detection threshold. *C *Carbon, *N* Nitrogen, *O* Oxygen, *Na* Sodium, *Mg* Magnesium, *P* Phosphorus, *Ca* Calcium, *Fe* Iron. *Ca/P* Calcium-to-phosphorus atomic ratio. *CTE* Conventional total-etch (35% phosphoric acid) applied to noncontaminated dentin, *MTE* Modified total-etch (1% gallic acid) applied to noncontaminated dentin, *FS_SE* No conditioning (Self-Etch) applied to ferric sulfate-contaminated dentin, *FS_CTE* Conventional total-etch applied to ferric sulfate-contaminated dentin, *FS_MTE* Modified total-etch applied to ferric sulfate-contaminated dentin

#### Residual iron detection

Iron (Fe) was detected only in FS-Contaminated specimens that did not undergo subsequent phosphoric acid conditioning. Specifically, FS_SE and FS_MTE groups exhibited quantifiable Fe content (0.57 ± 0.04 atomic% and 0.62 ± 0.13 atomic%, respectively), whereas CTE, MTE, and FS_CTE groups showed Fe content below the detection limit (< 0.01 atomic%). Phosphoric acid conditioning (FS_CTE) reduced iron to below the instrument detection limit (< 0.01 atomic%), whereas gallic acid conditioning (FS_MTE) retained measurable Fe deposits at levels comparable to those observed with water rinsing alone (FS_SE).

#### Mineral content analysis

Descriptive EDS analysis indicated differences in phosphorus and calcium content among the treatment protocols. MTE groups recorded higher mean P (5.36 ± 0.81 atomic%) and Ca (8.39 ± 1.14 atomic%) values compared to CTE (P: 0.77 ± 0.27%; Ca: 1.13 ± 0.37%) and FS_CTE groups (P: 1.15 ± 0.07%; Ca: 1.65 ± 0.11%). Groups contaminated with ViscoStat but not subsequently treated with phosphoric acid showed intermediate values: FS_SE (P: 4.14 ± 0.15%; Ca: 6.18 ± 0.24%) and FS_MTE (P: 3.75 ± 0.14%; Ca: 5.60 ± 0.18%).

The Ca/P atomic ratio was higher in MTE specimens (1.57 ± 0.07) than in CTE specimens (1.47 ± 0.02). Similarly, FS_MTE (1.49 ± 0.06) showed a higher ratio than FS_CTE (1.43 ± 0.02). The physiological dentin Ca/P ratio is approximately 1.67. These values descriptively indicate a trend toward a mineral profile closer to that of apatite-like dentin in the MTE groups. Given that the mineral phase of dentin is predominantly apatite-like and that its Ca/P ratio has been reported to approximate that of hydroxyapatite, such variation should be interpreted with caution, as reported values may differ depending on the substrate characteristics and analytical method used [42].

#### Organic matrix components

Carbon content differed descriptively across groups. CTE and FS_CTE recorded the highest mean C values (49.68 ± 0.93% and 48.37 ± 0.72%, respectively), whereas MTE recorded the lowest (29.29 ± 3.87%). Mean oxygen content was higher in MTE (45.03 ± 3.59%) than in CTE (27.36 ± 0.48%).

Descriptively, mean nitrogen content was higher in phosphoric acid-treated groups (CTE: 20.61 ± 0.28%; FS_CTE: 20.22 ± 0.33%) than in gallic acid-treated groups (MTE: 11.30 ± 1.74%; FS_MTE: 15.22 ± 0.54%). Although the pattern is consistent with greater collagen exposure following phosphoric acid conditioning, nitrogen detected by EDS at 15 kV may also originate from adventitious organic contamination, residual surface deposits, or organic matrix components unrelated to collagen; in the absence of an unconditioned baseline control and inferential statistical analysis, this interpretation cannot be confirmed.

#### Trace elements

Trace elements (Na, Mg, Al) were detected in low mean concentrations (< 0.35 atomic%) across all groups. Descriptively, MTE and FS_MTE recorded higher mean Na (0.31 ± 0.04% and 0.16 ± 0.05%, respectively) and Mg (0.33 ± 0.11% and 0.25 ± 0.02%) values than CTE (Na: 0.03 ± 0.02%; Mg: 0.05 ± 0.02%).

## Discussion

The present findings partially confirm and partially diverge from those of the prior investigation [14]. Consistent with prior work, gallic acid preconditioning (MTE) achieved bond strengths significantly exceeding those with self-etch application and supported adequate short-term dentin bonding. However, in contrast to the previously reported superiority of MTE over CTE under contamination, no statistically significant difference was detected between MTE and CTE in the overall protocol comparison (*p* = 0.591). This divergence likely reflects differences in contamination chemistry (ferric sulfate versus Ankaferd Blood Stopper), adhesive system composition, and the EDS characterization, which revealed that gallic acid retains rather than removes iron from contaminated surfaces. The central finding, that gallic acid retained measurable iron on ferric sulfate-contaminated dentin surfaces yet yielded mean bond strength values comparable to those of phosphoric acid conditioning, raises a mechanistically important question: how does comparable short-term bond strength arise from fundamentally different surface chemistries? To the author’s knowledge, no previous study has simultaneously characterized the mechanical, elemental, and morphological consequences of gallic acid preconditioning on ferric sulfate-contaminated dentin, and both research hypotheses were supported by converging evidence from all three analytical approaches. The novel contributions of the present study are: (1) EDS elemental confirmation that gallic acid retains, rather than removes, ferric sulfate-derived iron; (2) a ferric sulfate-specific contamination model; and (3) a dual-adhesive comparison across systems with distinct functional monomers and polymerization mechanisms.

The directional consistency of the present MTE findings with the most recent investigation in this area further supports the reproducibility of gallic acid’s preconditioning effect on noncontaminated dentin. Ghanbarian et al. (2025) reported a mean shear bond strength of 21.72 ± 4.12 MPa following 1% (w/v) gallic acid preconditioning prior to Gluma Bond Universal, a value closely comparable to the 20.80 ± 3.05 MPa observed in the present MTE groups [39]. This numerical concordance across independent investigations employing different universal adhesives reinforces the consistency of short-term bonding performance achievable with 1% gallic acid on uncontaminated substrates, and supports the rationale for retaining the 1% (w/v) concentration in the present study [39]. Neither prior investigation, however, incorporated a hemostatic contamination model or post-aging surface characterization, leaving the iron-retention behavior demonstrated here as a novel contribution.

The first research hypothesis was supported: three-way ANOVA revealed statistically significant main effects for all three independent variables (all *p* < 0.001; Table 3). Application protocol exerted the greatest influence (ηp² = 0.228), followed by adhesive type (ηp² = 0.166) and contamination condition (ηp² = 0.085). Both gallic acid and phosphoric acid conditioning significantly exceeded self-etch protocols (17.46 ± 3.19 MPa, *p* < 0.001), consistent with the literature documenting enhanced bonding following preconditioning of hemostatic agent-contaminated dentin [1, 11, 14, 43]. The absence of a significant MTE–CTE difference contrasts with the previously reported superiority of MTE under contamination [17], a discrepancy likely reflecting differences in contamination chemistry: ferric sulfate’s strongly acidic pH and iron deposition may represent a more chemically aggressive substrate modification than blood contamination, partially offsetting gallic acid’s advantage; however, this hypothesis requires direct experimental comparison and cannot be confirmed from the present data alone.

The second research hypothesis, that 1% (w/v) gallic acid preconditioning affects residual iron content on contaminated dentin surfaces, was descriptively supported by EDS and SEM observations, although the underlying chemical mechanism remains unconfirmed. Water rinsing alone (FS_SE: 0.57 ± 0.04 atomic%) and gallic acid conditioning (FS_MTE: 0.62 ± 0.13 atomic%) both retained measurable iron at descriptively similar levels, whereas phosphoric acid conditioning (FS_CTE: <0.01 atomic%) reduced iron to below the EDS detection limit. This retention pattern is consistent with gallic acid’s known affinity for Fe³⁺ through its ortho-dihydroxyl and galloyl moieties [37]; however, whether this reflects true chelation complex formation, physical entrapment, or incomplete removal cannot be determined from EDS data alone and warrants spectroscopic confirmation (e.g., XPS, FTIR, or Raman). SEM corroborated these findings: FS_MTE surfaces exhibited abundant fine, scattered precipitates (Fig. 7) morphologically distinct from the coarser crystalline aggregates observed in FS_SE specimens (Figs. 5), providing additional evidence consistent with a surface interaction between gallic acid and iron-containing residues. It should be noted that EDS was performed without certified reference standards or nonnconditioned baseline; consequently, differences in carbon and nitrogen content between groups should be interpreted with caution. Furthermore, EDS provides semi-quantitative rather than absolute elemental characterization. Within these constraints, the contrast between detectable iron in the FS_MTE group and non-detectable iron in the FS_CTE nonetheless supports the interpretation of differential iron retention following conditioning.

The clinical significance of retained iron-associated surface deposits warrants consideration. Theoretical concerns include potential interference with functional monomer–hydroxyapatite bonding and iron-mediated oxidative pathways; however, none of these mechanisms were directly assessed in the present study. Importantly, under the tested short-term aging conditions, these retained surface deposits did not appear to adversely affect short-term mechanical performance; however, this inference rests on the absence of a significant Contamination × Protocol interaction (*p* = 0.323) rather than a dedicated FS_MTE versus FS_CTE subgroup analysis, and should be interpreted accordingly. The superior performance of total-etch protocols (CTE, MTE) compared to self-etch (*p* < 0.001) aligns with the literature, which documents enhanced bonding following preconditioning of dentin contaminated with a hemostatic agent [1, 11, 14, 43].

That gallic acid and phosphoric acid conditioning yielded comparable overall bond strength despite fundamentally different mechanisms of action is noteworthy. Descriptive EDS profiles revealed higher mean Ca content and Ca/P ratios in MTE groups compared to CTE, consistent with greater mineral retention (Table 5). Higher mean C and N content in CTE groups is consistent with greater collagen exposure following phosphoric acid demineralization, though this interpretation is confounded by the absence of a nonconditioned baseline and multiple potential nitrogen sources detectable by EDS at 15 kV. The mineral preservation pattern observed with gallic acid may, in theory, support chemical adhesion through functional monomer–hydroxyapatite interactions; however, these potential advantages were not experimentally assessed and remain speculative.

Collectively, this surface-modification profile, greater mineral retention, conservative smear-layer modification, and documented MMP-inhibitory activity of galloyl-containing compounds in the literature [22, 25, 38] align with the properties expected of biointeractive conditioning agents that seek to complement immediate micromechanical retention with a biologically supportive substrate environment; whether these properties translate to long-term bonding advantages remains the central question arising from the present work.

Adhesive failures predominated across all groups (≥ 73.3%), indicating that fractures consistently occurred at the resin–dentin interface rather than within the dentin substrate. The complete absence of cohesive dentin failures suggests that the mechanical integrity of the dentin substrate was maintained under the testing conditions employed, irrespective of the preconditioning agent used. Because failure-mode distributions were not subjected to inferential statistical analysis, these observations are reported descriptively and should not be interpreted as evidence of differential clinical efficacy between protocols.

Ferric sulfate contamination systematically increased mixed failures (+ 6.7% to + 26.7%), with the most pronounced effect in gallic acid-treated groups (ABU-MTE: 0% vs. FS_ABU-MTE: 26.7%). Phosphoric acid preconditioning resulted in fewer mixed failures (FS_ABU-CTE: 13.3%), a pattern that may reflect more uniform interfacial behavior; however, no formal statistical comparison of failure mode distributions was performed. The discrepancy between the comparable overall bond strengths of MTE and CTE and the divergent failure distributions observed under ferric sulfate contamination is consistent with spatially heterogeneous surface chemistry at the gallic acid-conditioned interface: the concurrent EDS evidence of residual iron retention and the SEM observation of broadly distributed fine precipitates in the FS_MTE group suggest that localized iron complex accumulation may compromise resin infiltration in discrete areas, manifesting as mixed failures despite adequate overall mean bond strength. Whether this reflects differential resin penetration, altered surface wettability, or other interfacial factors cannot be determined without direct hybrid layer imaging. This heterogeneity may not affect short-term strength but could represent sites for progressive degradation under extended clinical loading and hydrolytic challenge; however, this interpretation is descriptive and requires validation in larger samples and under extended aging protocols.

It should be noted that bond strength values obtained under standardized in vitro conditions reflect relative differences between experimental groups rather than direct predictors of clinical performance, as laboratory testing employs operator-controlled, extraoral parameters that do not replicate the complexity of the intraoral environment. With this caveat, All Bond Universal demonstrated statistically higher bond strengths than Tokuyama Universal Bond (20.73 ± 3.05 vs. 18.31 ± 3.44 MPa, *p* < 0.001), likely reflecting fundamental compositional and mechanistic distinctions between the two systems.

ABU’s primary functional monomer, 10-MDP, has a well-established capacity to form stable ionic bonds with calcium through self-assembled nano-layering at the adhesive–hydroxyapatite interface [44–46], whether this property contributes differentially to bonding performance on mineral-preserved surfaces, such as those observed descriptively following gallic acid conditioning, was not assessed in the present study. TUB relies on a 3D-SR phosphoric acid ester monomer and MTU-6 for dentin bonding without 10-MDP [45, 47]. While the 3D-SR monomer forms ionic bonds with calcium through multiple phosphate groups [48], its performance on mineral-preserved substrates may differ from that of MDP-based systems. Beyond functional monomer differences, TUB’s reliance on chemical polymerization via a borate-based initiator system [48, 49] may render it more susceptible to surface contaminants and environmental variability than ABU’s light-cured mechanism, as chemical polymerization is inherently sensitive to moisture, oxygen inhibition, and surface contaminants that have minimal impact on light-activated systems [47, 50]; whether retained ferric ions specifically interfere with this pathway remains an open question. The lower pH of TUB (2.2 vs. ABU 3.2) may additionally influence calcium salt formation and monomer infiltration. The relative contribution of each factor cannot be determined from the present data, and these interpretations remain speculative.

As noted in the Methods, the TUB evaluated here was the original formulation; performance interpretations should not be extrapolated to 10-MDP-containing Tokuyama formulations. Tokuyama Universal Bond exhibited residual mixed failures even under noncontaminated conditions (6.7–20.0%), representing a baseline vulnerability that intensified under contamination. The combination of the absence of MDP (a monomer associated with calcium-binding capacity in MDP-containing systems), reliance on chemical polymerization, lower pH relative to ABU, and a two-component mixing system that introduces compositional variability and working time constraints absent in ABU’s single-component formulation may collectively contribute to TUB’s inferior bonding performance and elevated mixed-failure proportions under the tested conditions. However, as noted above, the relative contribution of each factor cannot be determined from the present data. Taken together, the failure mode distribution data suggest that when contamination is avoidable, both conditioning agents support consistent bonding outcomes. Following ferric sulfate exposure, however, phosphoric acid was associated with a lower proportion of mixed failures than gallic acid (FS_CTE: 13.3% vs. FS_MTE: 26.7%), suggesting more uniform interfacial behavior, though this observation remains descriptive.

The clinical oral environment imposes continuous biomechanical, thermal, and biochemical challenges on adhesive interfaces that cannot be fully replicated in short-term in vitro models. The present study employed 5,000 thermocycles as an approximation of short-term intraoral aging [40, 41]; however, the long-term survival of gallic acid-conditioned adhesive interfaces under physiologically relevant environmental stressors, including salivary enzyme activity, cyclic occlusal loading, and pH fluctuations, remains to be established. Whether the iron-associated surface deposits retained at gallic acid-treated surfaces remain stable or transform progressively under physiological conditions represents a critical gap between the comparable short-term mechanical performance demonstrated in the overall protocol analysis and the long-term clinical performance that ultimately determines restoration survival.

### Limitations

Several limitations must be openly acknowledged: Macro-shear bond strength testing was employed to enable direct methodological comparison with prior investigation using identical geometry [17] and with the hemostatic contamination literature [11–14]. However, macro-SBS yields more heterogeneous stress distributions than microshear or microtensile (µTBS) testing, potentially reducing sensitivity to localized bonding defects. In future studies, employing µTBS testing may enable a more sensitive detection of interfacial heterogeneity, which is particularly relevant given the spatially variable surface chemistry expected from the gallic acid–iron surface interaction. Artificial aging was limited to 5,000 thermocycles (5 °C/55°C), which were insufficient to evaluate long-term interface stability. SEM (*n* = 3) and EDS (*n* = 5) sample sizes preclude inferential analysis. Furthermore, EDS was performed without certified reference standards or a nonconditioned baseline, limiting interpretation of organic matrix components. Only one gallic acid concentration (1% w/v) was evaluated, and the in vitro design lacks pulpal pressure, dentinal fluid dynamics, salivary proteins, biofilm, and masticatory stresses. Only two adhesive systems were tested, limiting generalizability. Regarding study design, the split-tooth allocation, with the mesial half assigned to ABU and the distal half to TUB, functioned as a within-tooth-matched design, controlling for tooth-level variation in dentin quality and tubule morphology. Although this introduces within-tooth correlation for the adhesive type comparison, any residual correlation would act conservatively, reducing rather than inflating the F-statistic. The tooth was not modeled as a random effect, consistent with standard practice in dental bonding research; this limitation is acknowledged.

### Future directions

Future investigations should prioritize: (1) microtensile bond strength (µTBS) testing to enable more sensitive detection of interfacial heterogeneity at the gallic acid–iron modified interface; (2) long-term durability assessment using extended aging protocols (≥ 12 months water storage, mechanical cycling, and enzymatic challenge) to determine whether retained iron-associated surface deposits influence adhesive interface stability over clinically relevant time frames; (3) spectroscopic characterization (XPS, Raman, or FTIR) to confirm the coordination chemistry and stability of gallic acid–iron surface interactions under physiological pH conditions and in the presence of adhesive monomers; (4) transmission electron microscopy with elemental mapping to visualize iron distribution within the hybrid layer and to determine whether functional monomer nano-layering is established on gallic acid-conditioned mineral-preserved surfaces; (5) mechanism-based studies directly assessing MMP activity in hybrid layers formed under gallic acid versus phosphoric acid conditioning; (6) systematic dose-response studies (0.5–5% w/v) to optimize gallic acid concentration and application parameters; and (7) prospective randomized clinical trials with a minimum 3–5 years follow-up in contamination-prone clinical scenarios.

## Conclusions

This study provides the first combined mechanical, elemental, and morphological characterization of gallic acid as a preconditioning agent on ferric sulfate-contaminated dentin. Gallic acid produced a surface-modification profile fundamentally distinct from that of phosphoric acid: it retained iron-associated surface deposits rather than removing them, and yielded a more conservative smear layer modification. Despite these mechanistic contrasts, overall short-term bond strength did not differ significantly between the two conditioning agents. All Bond Universal demonstrated statistically higher overall mean bond strength than Tokuyama Universal Bond across all tested conditions.

Within the limitations of this in vitro design, the following evidence-based recommendations are supported by the present findings: Rubber dam isolation should be prioritized whenever clinically feasible. On noncontaminated dentin, gallic acid preconditioning may represent a biologically conservative alternative to phosphoric acid, preserving greater mineral content and offering a distinct surface profile; whether this profile translates to any bonding advantage under extended aging conditions, however, remains to be determined. Under ferric sulfate contamination, phosphoric acid conditioning is the better-supported choice: it eliminated detectable iron residue, produced no bond-strength disadvantage relative to gallic acid, and was associated with lower mixed-failure proportions. Gallic acid should not be recommended as a substitute for phosphoric acid in ferric sulfate-contaminated scenarios until long-term durability data from extended aging protocols and evidence from prospective clinical trials become available.

The influence of retained iron-associated surface deposits on adhesive interface stability under extended environmental challenges, and whether gallic acid’s mineral-preserving properties confer long-term bonding advantages, remain the central open questions that must be addressed before gallic acid can be adopted as a routine clinical alternative to phosphoric acid conditioning.

## Supplementary Information

Below is the link to the electronic supplementary material.

Supplementary Material 1 (DOCX 940 KB)

## Data Availability

The datasets generated and/or analyzed during the current study are available from the corresponding author on reasonable request.
