# Supplementary material for: Gallic acid as a biointeractive dentin conditioning agent for universal adhesives: bond strength, iron retention, and surface characterization following ferric sulfate hemostatic contamination
Source: Clin Oral Investig. 2026 Jun 26;30(7):301. doi: 10.1007/s00784-026-06987-5 (PMC13303769; doi:10.1007/s00784-026-06987-5)
Supplement: Supplementary file 1 — Supplementary Material 1 (DOCX 940 KB) [file 784_2026_6987_MOESM1_ESM.docx]

**Supplementary Material**

*Gallic Acid as a Biointeractive Dentin Conditioning Agent for Universal Adhesives:*

*Bond Strength, Iron Retention, and Surface Characterization Following Ferric Sulfate Hemostatic Contamination*

**Supplementary Figure S1.** Individual Shear Bond Strength Values with Group Means ± SD (n = 15 per group; 12 groups; 180 specimens total)


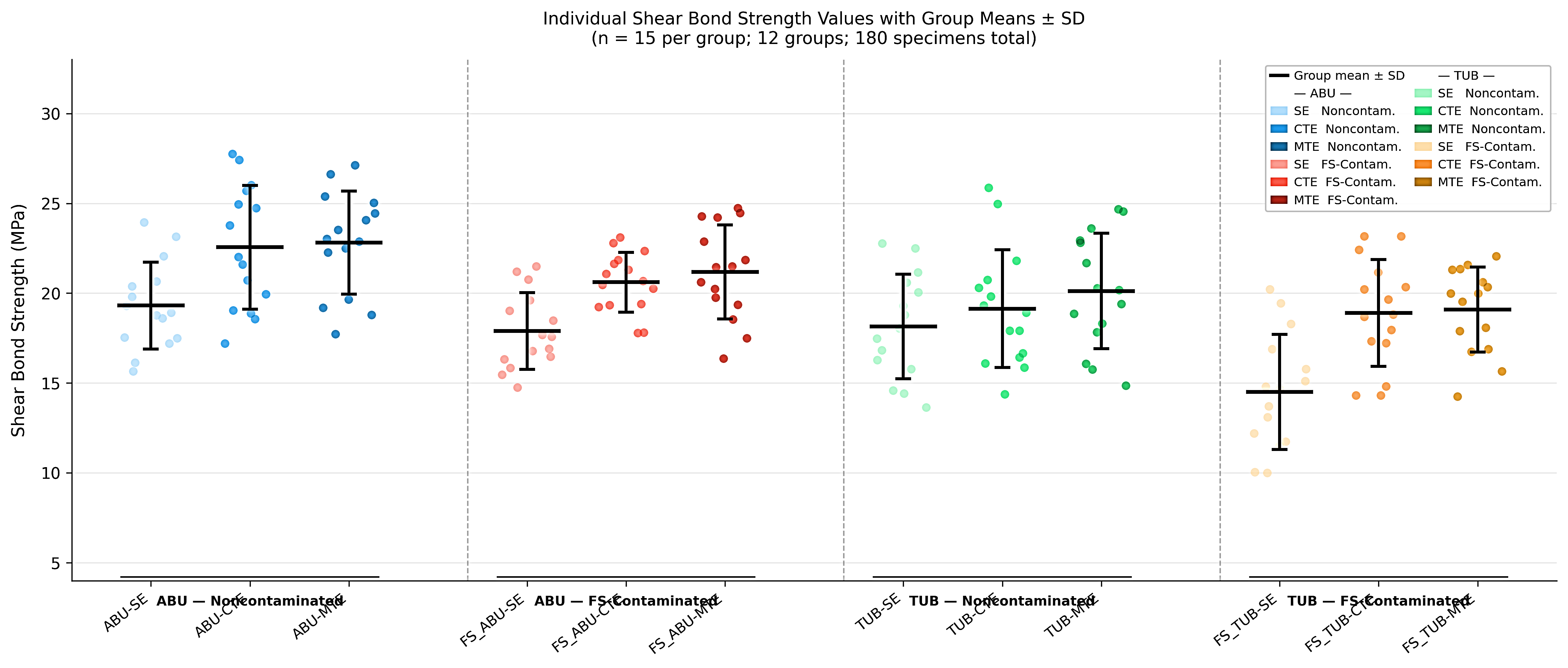


FS_TUB-SE FS_TUB-CTE FS_TUB-MTE

FS_ABU-SE FS_ABU-CTE FS_ABU-MTE

TUB-SE TUB-CTE TUB-MTE

ABU-SE ABU-CTE ABU-MTE

ABU – Noncontaminated

ABU – FS Contaminated

TUB – FS Contaminated

TUB – Noncontaminated

**Figure Notes**

**Data representation.** Each data point represents the shear bond strength (SBS) of a single, bonded specimen (a composite cylinder bonded to dentin). Horizontal crossbars represent group means; vertical bars represent ±1 standard deviation (SD). All 180 individual SBS values are plotted (n = 15 per group, 12 groups).

**Group organization.** Groups are organized by adhesive system (All Bond Universal [ABU] vs. Tokuyama Universal Bond [TUB]) and dentin contamination condition (Noncontaminated vs. Ferric Sulfate [FS]-Contaminated). Within each combination, three adhesive application protocols are shown: SE = Self-Etch; CTE = Conventional Total-Etch (35% phosphoric acid); MTE = Modified Total-Etch (1% [w/v] gallic acid). Circle markers (○) denote noncontaminated groups; square markers (□) denote ferric sulfate-contaminated groups.

**Statistical analysis.** A three-way analysis of variance (ANOVA) revealed statistically significant main effects of contamination condition (F[1,168] = 15.612, p < 0.001, η*p*² = 0.085), adhesive type (F[1,168] = 33.345, p < 0.001, η*p*² = 0.166), and adhesive application protocol (F[2,168] = 24.761, p < 0.001, η*p*² = 0.228). No statistically significant two-way or three-way interaction effects were detected (all p > 0.05). Post-hoc pairwise comparisons (Tukey’s HSD) revealed that the SE protocol yielded significantly lower mean SBS than both CTE (mean difference = 2.84 MPa, 95% CI [1.63, 4.05], p < 0.001) and MTE (mean difference = 3.34 MPa, 95% CI [2.13, 4.55], p < 0.001); no significant difference was detected between CTE and MTE (mean difference = 0.50 MPa, p = 0.591). The model explained 38.4% of total variance (R² = 0.384, adjusted R² = 0.344).

**Statistical assumptions.** Data normality was confirmed individually for all 12 groups by the Shapiro-Wilk test (W > 0.93, p > 0.05 for all groups). Homogeneity of variances was verified by Levene’s test (F[11,168] = 1.305, p = 0.225). Both parametric ANOVA assumptions were satisfied. No specimens were excluded from the analysis.

**Abbreviations.** ABU = All Bond Universal (Bisco Inc., Schaumburg, IL, USA); TUB = Tokuyama Universal Bond (Tokuyama Dental Corp., Tokyo, Japan); SE = Self-Etch; CTE = Conventional Total-Etch (35% phosphoric acid); MTE = Modified Total-Etch (1% [w/v] gallic acid preconditioning); FS = Ferric Sulfate-Contaminated (ViscoStat, 20% ferric sulfate); NC = Noncontaminated; SD = Standard Deviation; SBS = Shear Bond Strength; ANOVA = Analysis of Variance; HSD = Honestly Significant Difference; CI = Confidence Interval.
